# An uncommon case of neonatal asphyxia associated with infantile-onset Pompe disease

**DOI:** 10.1186/s13052-025-02088-3

**Published:** 2025-08-22

**Authors:** Francesco Leo, Luca Barchi, Giulia Russo, Eleonora Balestri, Elena Chesi, Francesco Di Dio, Livia Garavelli, Lorenzo Iughetti, Giancarlo Gargano

**Affiliations:** 1 Neonatal Intensive Care Unit, Azienda USL-IRCCS di Reggio Emilia, 42123 Reggio Emilia, Italy; 2 Postgraduate School of Pediatrics, Department of Medical and Surgical Sciences of the Mother, Children, and Adults, University of Modena and Reggio Emilia, 41125 Modena, Italy; 3 Medical Genetics Unit, Department of Mother and Children, Azienda USL-IRCCS di Reggio Emilia, 42123 Reggio Emilia, Italy

**Keywords:** Asphyxia, Leonatal, Pompe disease, Heart disease, Newborn screening

## Abstract

**Background:**

Pompe disease, also known as glycogenosis type II or acid maltase deficiency, is an autosomal recessive disease caused by a deficiency of alpha-glucosidase. The severity depends mainly on the type of mutation, which in turn determines early or late onset; therapy modifies the outcome but does not alter the severity of the disease at presentation.

**Case presentation:**

We present a case report of a male infant, inborn and delivered at a gestational age of 39 weeks. Medical history reveals consanguineous parents with no invasive screening tests performed during pregnancy. They chose not to undergo prenatal screening even though they were aware of the risks associated with their consanguinity. At birth, the newborn was atonic and pale, with a heart rate of 70 bpm. During resuscitation, an umbilical venous catheter was placed, and three doses of adrenaline and one dose of bicarbonate were administered. At the Neonatal Intensive Care Unit, he underwent therapeutic hypothermia. Echocardiography, performed a few hours later, revealed severe biventricular and septal hypertrophy consistent with non-obstructive hypertrophic cardiomyopathy. During recovery, even after the discontinuation of hypothermia, the newborn exhibited abnormal neurological signs, including axial hypotonia and a tendency to keep his mouth open with tongue protrusion. Given the clinical picture and the early detection of septal and biventricular hypertrophy, genetic testing was performed, revealing a homozygous c.2560 C > T variant in the acid alpha-glucosidase gene (both parents were carriers), described in scientific literature as a class 5 pathogenic variant associated with glycogenosis type II (Pompe disease).

**Conclusion:**

Pompe disease is a rare genetic disorder and may be difficult to diagnose at birth. Suspicion should arise in the presence of hypertrophic cardiomyopathy, especially when associated with a history of neonatal asphyxia and abnormal neurological signs. An accurate diagnosis and early treatment are essential to improving the patient’s survival and quality of life.

## Background

Pompe disease (PD) (OMIM: 232300), also known as acid alpha-glucosidase (GAA) deficiency or glycogen storage disease type II (GSD II), is a rare, severe, progressive, autosomal recessive disorder. The underlying genetic defect is a pathogenic variant in the gene encoding lysosomal acid alpha-1,4-glucosidase, located at 17q25.2-q25.3, which disrupts lysosomal degradation of glycogen, leading to its accumulation within lysosomes [1]. This defect affects respiratory, cardiac, skeletal, and smooth muscle tissues [2]. The incidence of GSD II is estimated at 1 in 40,000 live births [2]. A recent study assessed the incidence in Italy to be 1/18,795 (IOPD 1/68,914; LOPD 1/25,843) [3]. Pompe disease is usually classified into two types: early (infantile, classic) and late-onset. Early-onset disease typically presents within the first months of life (median age: four months) and is characterized by cardiomegaly, respiratory distress, muscle weakness, and feeding difficulties; it represents the most severe form [4]. The late-onset form can appear at any age, is usually not associated with cardiomyopathy, and is generally less severe [5]. The purpose of this case report is to highlight an instance of early-onset Pompe disease associated with neonatal asphyxia. These data may help clinicians recognize the characteristic features, enable early diagnosis, and initiate enzyme replacement therapy (ERT) promptly to decrease the morbidity and mortality associated with this disease.

## Case report/case presentation

A male Pakistani infant, born at a gestational age of 39 weeks and 2 days with a birth weight of 3,070 g, was inborn. The pregnancy was reported to be uneventful. The parents were consanguineous, and no invasive screening tests were performed during pregnancy. At the obstetric evaluation in the delivery room, the amniotic fluid was found to be at the upper limit of normal, with a pathological umbilical artery pulsatility index (PI) and diastolic flow. The maternal vaginal-rectal swab was negative for GBS. At birth, the newborn was atonic and pale. He was stimulated, the upper airways were suctioned, and he was dried. Then, due to asystole on cardiac auscultation, intubation and ventilation were initiated. After 45 s of persistent asystole, cardiac massage was started and ventilation continued. A first dose of adrenaline was administered via the endotracheal tube (ETT) at 6 min of life. An umbilical venous catheter was placed, and a second dose of adrenaline was administered, followed by bicarbonate and a third dose of adrenaline. Asystole persisted at 10 min of life. At 15 min, heart rate and spontaneous respiratory activity appeared. Apgar scores were: 1 min– 0; 5 min– 0; 10 min– 0; 15 min– 4; 20 min– 4. The newborn was then extubated and transferred to the Neonatal Intensive Care Unit (NICU) in passive hypothermia, breathing spontaneously without respiratory support. Umbilical arterial blood gas analysis revealed mild metabolic acidosis. On physical examination, the newborn showed signs of moderate to severe hypoxic-ischemic encephalopathy (marked axial hypotonia, mild limb hypotonia, poorly elicited reflexes, and generalized hypomotility). Given the perinatal history and abnormal findings on cerebral function monitoring, therapeutic hypothermia was initiated at approximately 1 h of life.

**Fig. 1:**
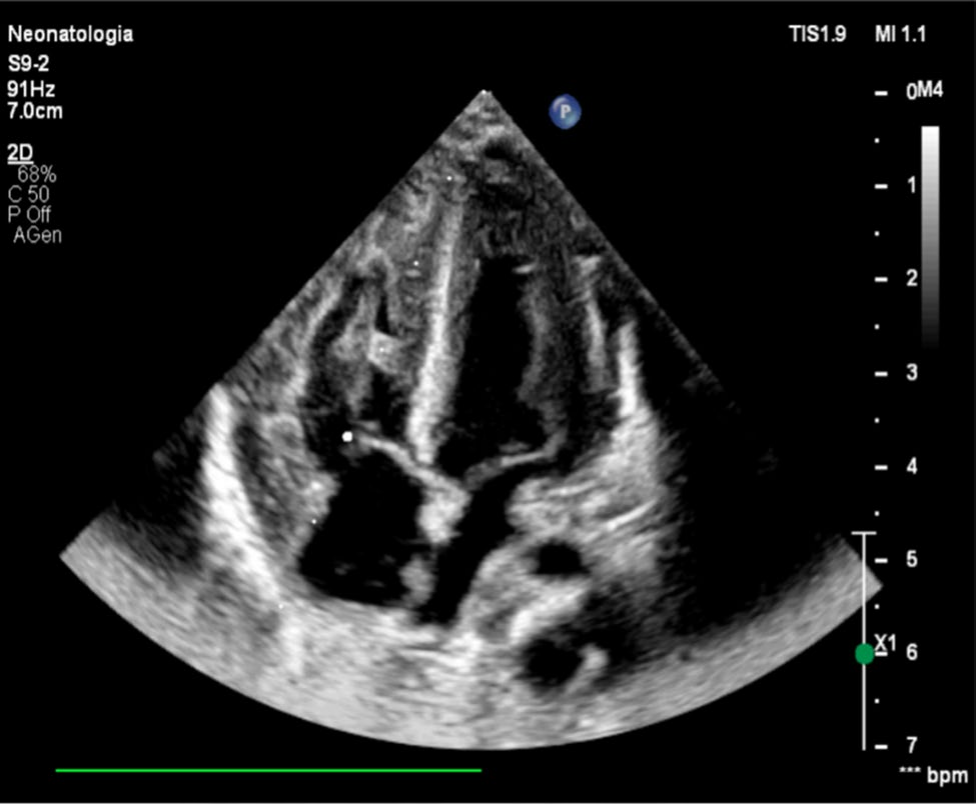
Four-chamber view showing biventricular hypertrophy

**Fig. 2:**
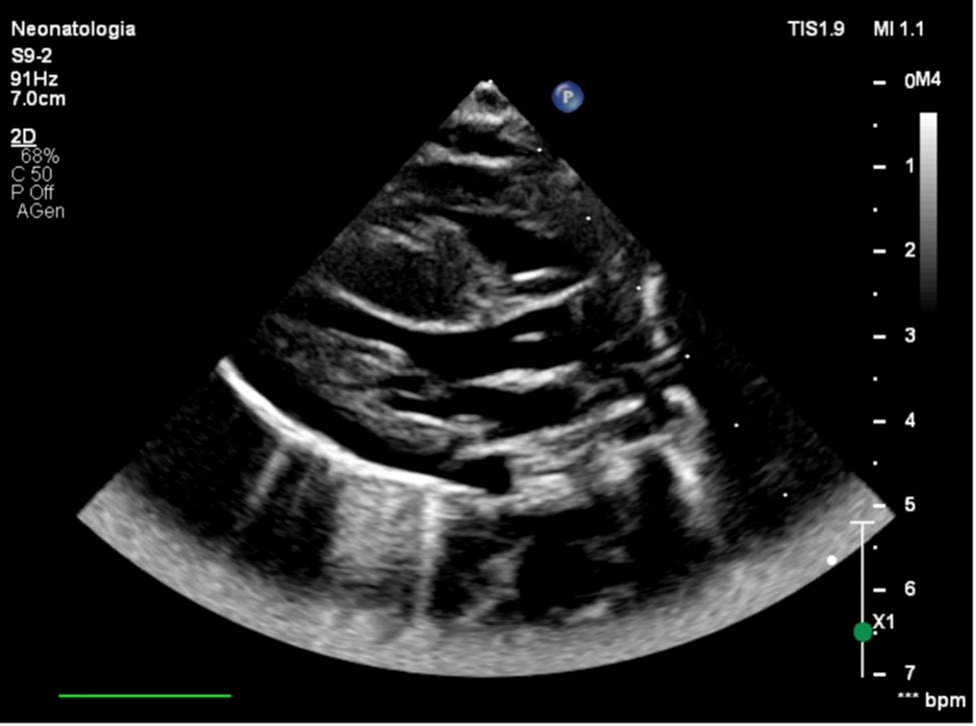
Long-axis view showing left ventricular hypertrophy, with prominent septal hypertrophy in the absence of left ventricular outflow tract (LVOT) obstruction

Laboratory tests on the first day of life revealed aspartate aminotransferase of 347 U/L (normal: 4–49 U/L), alanine aminotransferase of 69 U/L (normal: 2–40 U/L), lactate dehydrogenase of 427 U/L (normal: 120–216 U/L), troponin of 2797.5 ng/L (normal: 0–57.3 ng/L). Screening echocardiography, performed a few hours after birth, showed severe biventricular and septal hypertrophy with preserved contractility. IVSd measured 11 mm (Z-score: +4.7), and LVPWd measured 8 mm (Z-score: +4.7). An atrial septal defect was incidentally noted (Figs. 1 and 2).

**Fig. 3:**
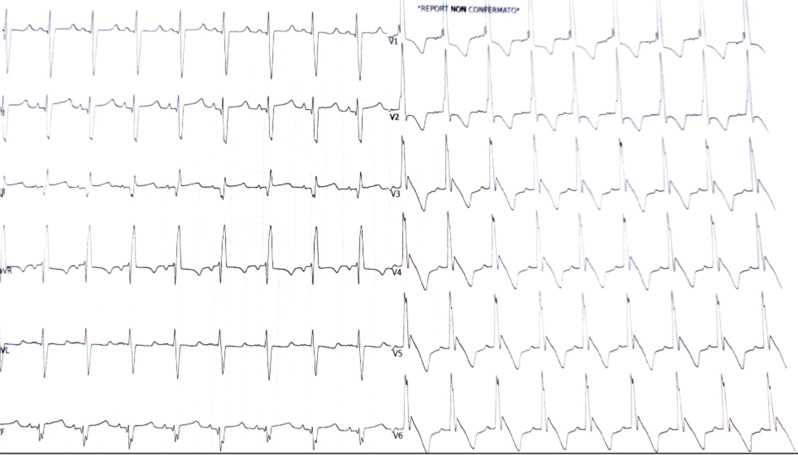
Electrocardiogram showing sinus rhythm with initial signs of right ventricular overload, including prominent R waves in leads V1 and V2, ST-segment changes, right bundle branch block, and a prolonged corrected QT interval (QTc)

**Fig. 4:**
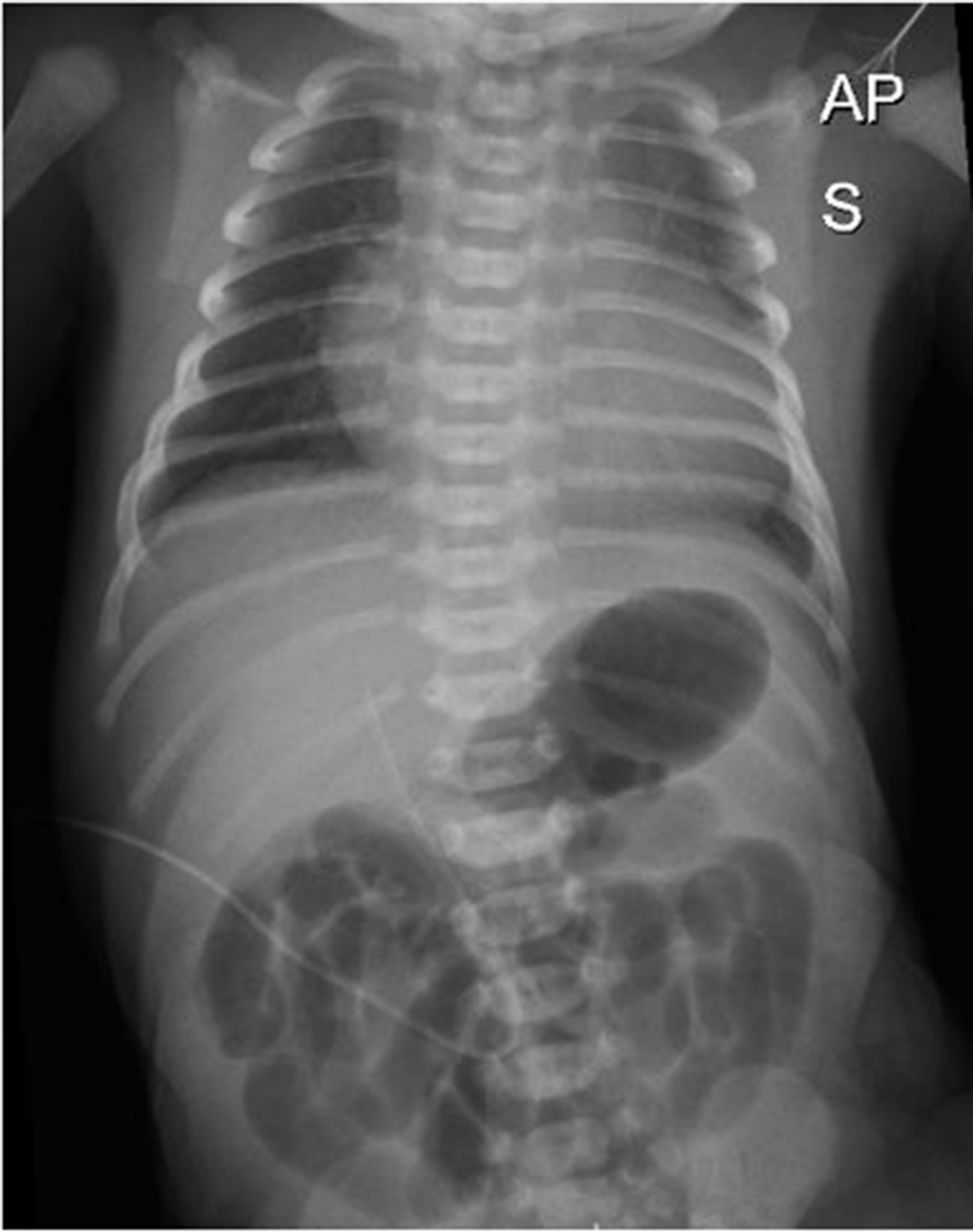
Chest X-Ray confirming severe cardiomegaly

Electrocardiogram (ECG) and chest X-ray were also performed, confirming ventricular hypertrophy (Figs. 3 and 4) with a cardiac index of 0.69.

During hospitalization, even after discontinuation of hypothermia, the newborn exhibited a neurological profile characterized by axial and perioral hypotonia, a tendency to keep his mouth open with tongue protrusion, weak and easily fatigable sucking, and diminished neonatal and palmar reflexes. Due to a tendency towards oxygen desaturation (SatO₂ deflection), the newborn required minimal oxygen support from the first days of life. This support was discontinued at around 15 days of life as vital signs stabilized, and he maintained spontaneous breathing in room air. Brain ultrasound monitoring and magnetic resonance imaging (MRI) were performed and yielded normal results. Follow-up echocardiographic monitoring confirmed persistent cardiac hypertrophy. Extended blood testing during recovery—including creatine phosphokinase, blood count, and electrolytes—was within normal limits. However, urinary glucose tetrasaccharide remained persistently elevated, with a mean value of 80 mmol/mol of creatinine. Given the clinical presentation and early detection of septal and biventricular hypertrophic cardiomyopathy suggestive of a storage disorder, blood samples were sent for analysis of acylcarnitines, sialotransferrin, very long-chain fatty acid (VLCFA), plasma amino acids, and blood sampling for whole-exome sequencing (WES). Genetic testing identified a homozygous variant (NM_000152.5(GAA): c.2560 C > T), with both parents found to be carriers. This variant is described in the scientific literature as a class 5 pathogenic mutation associated with glycogen storage disease type II (Pompe disease). Therefore, at 30 days of life, the newborn was transferred to a specialized hospital to initiate early enzyme replacement therapy.

## Discussion and conclusions

Pompe disease, also known as acid maltase deficiency or glycogen storage disease type II (GSDII), is an inherited, autosomal recessive disorder of glycogen metabolism caused by a deficiency of the lysosomal enzyme acid alpha-1,4-glucosidase (GAA). The structural gene encoding GAA is located on chromosome 17q25.2-q25.3 and consists of 20 exons, the first of which is non-coding. To date, more than 289 variants have been listed in the Pompe disease mutation database (www.pompecenter.nl), of which 197 have been demonstrated to be pathogenic [6]. GAA deficiency affects all cell types, but the most marked involvement is seen in cardiac, skeletal, and smooth muscle cells. The resulting accumulation of glycogen disrupts cellular architecture and contributes to progressive tissue damage. Pompe disease is classified into two clinical phenotypes: classic infantile-onset, in which symptoms appear before 1 year of age and are associated with hypertrophic cardiomyopathy; and late-onset, in which symptoms develop at or after 1 year of age and occur without hypertrophic cardiomyopathy [7]. The infantile form has a median age of onset of 2.4 (range 0.0–12.0 months) [8], and is characterized by severe hypotonia, progressive weakness, massive cardiomegaly, and variable hepatomegaly and macroglossia. Approximately 75% of patients with classic infantile-onset Pompe disease die before 12 months of age, with a median age at death of 8.7 months [9].

In this report, we describe a child with infantile-onset Pompe disease with clinical manifestations evident at birth. The c.2560G > T (p.Arg854X) mutation in exon 18 is typical of infantile-onset cases and appears to be more prevalent in Brazil and among Pompe patients of African descent [10].

The distinctiveness of the case we describe lies in the extremely early onset of symptoms, which at birth closely mimicked hypoxic-ischemic encephalopathy (HIE). HIE is a specific diagnosis that applies only when a neonate has encephalopathy known or strongly suspected to be caused by a hypoxic-ischemic event. The term *birth asphyxia*, which refers to impaired placental perfusion and gas exchange resulting in hypoxia, ischemia, and acidosis, was historically used to describe what is now HIE [5, 11, 12]. Current guidelines from the American College of Obstetricians and Gynecologists, endorsed by the American Academy of Pediatrics, recommend using the term HIE specifically in cases with clear evidence of an acute peripartum or intrapartum event. In our clinical case, the newborn’s condition appeared immediately critical, requiring tracheal intubation and the administration of three doses of adrenaline and one dose of bicarbonate. He also required respiratory support from the first hours of life until day 15 of life. Our patient met al.l clinical criteria for severe HIE and therefore underwent therapeutic hypothermia.

Cardiac hypertrophy, along with associated ECG and laboratory abnormalities, was initially and erroneously attributed to neonatal asphyxia and the need for advanced resuscitation with cardiac massage at birth. However, the persistence of a generalized hypotonia and feeding difficulties following hypothermic treatment—combined with the absence of MRI findings typically associated with asphyxial injury, and the continued presence of ventricular hypertrophy and macroglossia—prompted consideration of a differential diagnosis involving neuromuscular disorders and metabolic diseases.

Because inborn errors of metabolism can present during the neonatal period with neurologic distress, metabolic acidosis, and multiorgan involvement—features that resemble HIE— an underlying metabolic disorder may go undiagnosed unless the clinician maintains a high index of suspicion and performs appropriate diagnostic testing. Although individually rare, inborn errors of metabolism collectively affect approximately 1 in 1,000 neonates, warranting their inclusion in the differential diagnosis of newborns presenting with nonspecific signs suggestive of asphyxia. It is well established that such disorders may present in neonates with clinical features overlapping those of HIE [8]. Most neonates with metabolic disorders are born after an unremarkable delivery and experience an asymptomatic period lasting hours to days or longer. The onset of metabolic symptoms typically occurs postnatally, following a seemingly normal pregnancy and initial adaptation to extrauterine life. However, the absence of an initial symptom-free period does not rule out a metabolic disorder, as environmental stressors—such as traumatic delivery or prematurity—may unmask an underlying inborn error of metabolism. This phenomenon is often described as “double-trouble,” and HIE mimics should be considered when there is discordance between the clinical history, examination findings, laboratory results, imaging features, and clinical course [13].

The primary differential diagnosis included glycogen storage disease type IIB (Danon disease), congenital syndromes such as Noonan syndrome and Beckwith-Wiedemann syndrome, mitochondrial and respiratory chain disorders, spinal muscular atrophy type 1, GSD type IIIa, specific fatty acid oxidation disorders, and iatrogenic disorders [11–14]. Our patient did not exhibit metabolic abnormalities such as hypoglycemia or acidosis. Therefore, Pompe disease became the primary diagnostic consideration and was confirmed in the third week of life.

Based on the clinical course observed at birth, we hypothesize a prenatal onset of Pompe disease in our patient. Intrauterine onset of Pompe disease is extremely rare; only eight other cases have been reported in the literature [15]. Despite the term “*infantile*” in its classification, the disease can, in fact, manifest before birth. In the review by Xi et al. [16], all neonates showed myocardial abnormalities on intrauterine ultrasound, although the specific findings varied and included hypertrophic cardiomyopathy, dilated cardiomyopathy, and myocardial masses. In all reported cases, the condition progressed to myocardial hypertrophy postnatally. Laboratory markers, such as myocardial enzymes and B-type natriuretic peptide (BNP), were also elevated to varying degrees.

The possibility of prenatal diagnostics has been described in the literature in cases of clinical suspicion or a suggestive family history, allowing for the initiation of in utero enzyme-replacement therapy (ERT), or even standard postnatal treatment for the first days of life. Crucial to this approach is the presence of clinical suspicion and the timely establishment of a definitive diagnosis [5]. In our case, no elements of suspicion were present on the prenatal ultrasound scans, which were performed in Pakistan, where the pregnancy was monitored until the month before delivery. As a result, the diagnosis was delayed due to the overlap of asphyxia-like symptoms with those of early-onset infantile Pompe disease.

Enzyme replacement therapy (ERT) was initiated one week after diagnosis. According to the literature, symptoms of infantile-onset Pompe disease (Infantile-PD) typically emerge around two months of age, with diagnosis usually occurring around four months. Diagnosis within the first months of life is rare [1, 6, 7]. Classic infantile-onset Pompe disease progresses rapidly without treatment, with a median survival of one to two years [1, 6, 7]. For this reason, some countries have implemented newborn screening (NBS) programs for Pompe disease. For example, the United States Advisory Committee on Heritable Disorders in Newborns and Children recommended the inclusion of GAA deficiency in the Recommended Uniform Screening Panel (RUSP). NBS for Pompe disease typically follows a two-stage algorithm [5]. The first stage involves analysis of GAA activity in dried blood spots, using methods such as digital microfluidics, fluorometric assays, or tandem mass spectrometry [5, 11]. The second stage typically includes molecular genetic testing [5, 11]. In many countries, NBS for Pompe disease has not been adopted due to a high rate of false positives and uncertainty regarding the management of asymptomatic cases [12]. To date, Pompe disease is not included in Italy’s newborn metabolic screening program. Kronn et al. proposed a management protocol for asymptomatic infants diagnosed through screening. They recommend clinical monitoring that includes physical examination, ECG, echocardiography, brainstem auditory evoked responses, and developmental assessments—initially every three months during the first year of life, then annually thereafter [17]. ERT remains the cornerstone of treatment. Numerous studies in the literature have demonstrated its efficacy in improving cardiomyopathy, reducing the need for invasive ventilation, and enhancing motor development and function [14, 15].

On the other hand, osteopenia, dysphagia with aspiration risk, and arrhythmias such as Wolff-Parkinson-White syndrome may persist despite treatment [16]. Another critical aspect of treatment outcomes in children with infantile-onset GAA deficiency is the risk of immunogenicity-related complications. To mitigate this, combinations of immunomodulatory drugs—such as rituximab, methotrexate, and intravenous immunoglobulin—are often required [18]. Emerging therapies, including gene therapy, are currently under investigation in phase I and II clinical trials. For example, the study by Salabarria et al. described the potential of gene therapy and its low risk of immunogenic complications [15].

In conclusion, Pompe disease is a severe, progressive, and rare disorder that can manifest in utero or during the first days of life. The overlap of its initial symptoms with other more common neonatal conditions—such as asphyxia, encephalopathy, and generalized hypotonia—can lead to delayed diagnosis.

In case of suspicion, it is necessary to promptly perform enzyme and genetic testing for early diagnosis and treatment. In the next few years, new gene therapy could emerge and completely change the prognosis for this disease. Is it time for universal NBS for Pompe disease?

## Data Availability

The datasets used and/or analyzed during the current study are available from the corresponding author upon reasonable request.

## Abbreviations

PD: Pompe disease
GAA: Acid alpha-glucosidase
GSD II: Glycogen storage disease type II
NICU: Neonatal intensive care unit
ECG: Electrocardiogram
MRI: Magnetic resonance imaging
HIE: Hypoxic-ischemic encephalopathy
VLCFA: Very long-chain fatty acid
WES: Whole-exome sequencing
ERT: Enzyme replacement therapy
NBS: Newborn screening
RUSP: Recommended uniform screening panel
